# FEA-Based Stress–Strain Barometers as Forecasters for Corneal Refractive Power Change in Orthokeratology

**DOI:** 10.3390/bioengineering11020166

**Published:** 2024-02-09

**Authors:** Lo-Yu Wu, Wen-Pin Lin, Richard Wu, Lynn White, Ahmed Abass

**Affiliations:** 1 Department of Power Mechanical Engineering, Nation Tsing Hua University, Hsinchu 300, Taiwan; 2 Research and Development Center, Brighten Optix Corporation, Taipei 111, Taiwan; 3 Department of Optometry, University of Kang Ning, Taipei 114, Taiwan; 4 College of Optometry, Pacific University, Forest Grove, OR 97116, USA; 5 Research and Development Department, LWVision, Leicester LE18 1DF, UK; 6 Department of Materials, Design and Manufacturing Engineering, School of Engineering, University of Liverpool, Liverpool L69 3GH, UK

**Keywords:** eye, cornea, Ortho-K, contact lenses, refractive power change, FEA

## Abstract

Purpose: To improve the effectivity of patient-specific finite element analysis (FEA) to predict refractive power change (RPC) in rigid Ortho-K contact lens fitting. Novel eyelid boundary detection is introduced to the FEA model to better model the effects of the lid on lens performance, and stress and strain outcomes are investigated to identify the most effective FEA components to use in modelling. Methods: The current study utilises fully anonymised records of 249 eyes, 132 right eyes, and 117 left eyes from subjects aged 14.1 ± 4.0 years on average (range 9 to 38 years), which were selected for secondary analysis processing. A set of custom-built MATLAB codes was built to automate the process from reading Medmont E300 height and distance files to processing and displaying FEA stress and strain outcomes. Measurements from before and after contact lens wear were handled to obtain the corneal surface change in shape and power. Tangential refractive power maps were constructed from which changes in refractive power pre- and post-Ortho-K wear were determined as the refractive power change (RPC). A total of 249 patient-specific FEA with innovative eyelid boundary detection and 3D construction analyses were automatically built and run for every anterior eye and lens combination while the lens was located in its clinically detected position. Maps of four stress components: contact pressure, Mises stress, pressure, and maximum principal stress were created in addition to maximum principal logarithmic strain maps. Stress and strain components were compared to the clinical RPC maps using the two-dimensional (2D) normalised cross-correlation and structural similarity (SSIM) index measure. Results: On the one hand, the maximum principal logarithmic strain recorded the highest moderate 2D cross-correlation area of 8.6 ± 10.3%, and contact pressure recorded the lowest area of 6.6 ± 9%. Mises stress recorded the second highest moderate 2D cross-correlation area with 8.3 ± 10.4%. On the other hand, when the SSIM index was used to compare the areas that were most similar to the clinical RPC, maximum principal stress was the most similar, with an average strong similarity percentage area of 26.5 ± 3.3%, and contact pressure was the least strong similarity area of 10.3 ± 7.3%. Regarding the moderate similarity areas, all components were recorded at around 34.4% similarity area except the contact pressure, which was down to 32.7 ± 5.8%. Conclusions: FEA is an increasingly effective tool in being able to predict the refractive outcome of Ortho-K treatment. Its accuracy depends on identifying which clinical and modelling metrics contribute to the most accurate prediction of RPC with minimal ocular complications. In terms of clinical metrics, age, Intra-ocular pressure (IOP), central corneal thickness (CCT), surface topography, lens decentration and the 3D eyelid effect are all important for effective modelling. In terms of FEA components, maximum principal stress was found to be the best FEA barometer that can be used to predict the performance of Ortho-K lenses. In contrast, contact pressure provided the worst stress performance. In terms of strain, the maximum principal logarithmic strain was an effective strain barometer.

## 1. Introduction

Orthokeratology (Ortho-K) is the contact lens practice of purposely reshaping the central corneal area to achieve a temporary reduction in refractive error. Originating in the 1950s, the modern Ortho-K lens design is reverse geometry and is manufactured from rigid materials with high oxygen permeability, allowing lenses to be worn overnight so that the wearer can see comfortably without refraction correction during the day [1,2,3]. Ortho-K lens design can vary slightly between brands, but typically, the lens is constructed so that the central back optic zone (BOZ) is followed by a reverse curve which then connects to one or more alignment zones. For myopia correction, the BOZ flattens the central cornea, effectively reducing its dioptric power, and the related area on the cornea has been defined as the Treatment Zone (TZ) [4,5]. In addition to simply reducing myopic refractive error, Ortho-K contact lenses are also increasingly used for myopia management and control [6,7,8]. FEA modelling to predict outcomes will become more important in the future.

The reverse curve in Ortho-K lenses creates a void between the posterior lens surface and the anterior corneal surface, allowing it to fill with tears forming a pressure gradient—positive pressure centrally and negative pressure peripherally. This results in epithelial migration from the central cornea to an annular, peripheral steepening zone (PSZ) corresponding to the reverse curvature position, thus initiating peripheral corneal thickening [2,9,10]. It is thought that the refractive effect of this PSZ may influence myopia control [11,12]. Understanding the biomechanics of the interaction between the lens, cornea, and eyelid may assist in designing Ortho-K contact lenses that can target refractive power change (RPC) more reliably.

Ortho-K treatment is monitored using topography machines where the TZ and the PSZ can be determined using tangential and axial curvature differential maps in addition to refractive power differential maps, comparing the treated corneal area to the baseline measurements. Although many topography machines now include fitting guidance for Ortho-K contact lenses, it would be useful if clinicians could also predict the outcome of Ortho-K in terms of RPC or indicate potential fit complications. Finite Element Analysis (FEA) is an emerging tool that can be used to simulate the behaviour of contact lenses on the eye [13,14,15]; however, the use of FEA to understand the mechanism of Ortho-K lenses on the eye is quite limited. Wu et al. investigated the cornea’s response to orthokeratology treatment via FEA simulation which showed that FEA stresses in Ortho-K correlate well with corneal curvature. However, it was not clinically validated [13] and Zhao et al. conducted a more detailed simulation-based FEA where stresses and displacements were investigated. The study confirmed the association between the displacement and differential power maps but without full clinical validation [16]. Both studies disregarded Ortho-K lens decentration entirely, substantially limiting their study results.

Although FEA incorporates such ocular attributes as tissue properties, Intra-ocular pressure (IOP), corneal curvature, and simulation of tear effects, applying eyelid properties is more complex. This study introduces a novel concept of identifying the eyelid margin via topography to allow the modelling of eyelid pressure effects on the eye and lens, thus increasing the accuracy of FEA.

The current study presents a wide-scale, patient-specific, clinically validated FEA utilising topography data of 249 eyes measured pre- and post-Ortho-K wear by a Medmont E300 topographer (Medmont International, Nunawading, Australia). A complete set of custom-built MATLAB 2023b (The MathWorks Inc., Natick, MA, USA) and Python 3.12.2 (Python Software Foundation, Wilmington, DE, USA) were used in parallel with Abaqus 6.14 (Dassault Systèmes, Vélizy-Villacoublay, France) FEA software to run the simulation. A master code written in MATLAB fully controlled the computing processes, including reading the topographer files and associated lens design data, building the FEA modelling components, scripting files, triggering Abaqus and Python software packages, reading the simulation output files and generating final results reports without human intervention. The output components of contact pressure, Mises stress, pressure, maximum principal stress and the strain output component of maximum principal logarithmic strain were investigated as possible metrics for Ortho-K contact lens performance on the eye regarding RPC; hence, the most correlated stress and strain barometers were identified.

## 2. Methods

### 2.1. Contact Lens Design and Manufacturing

**Figure 1:**
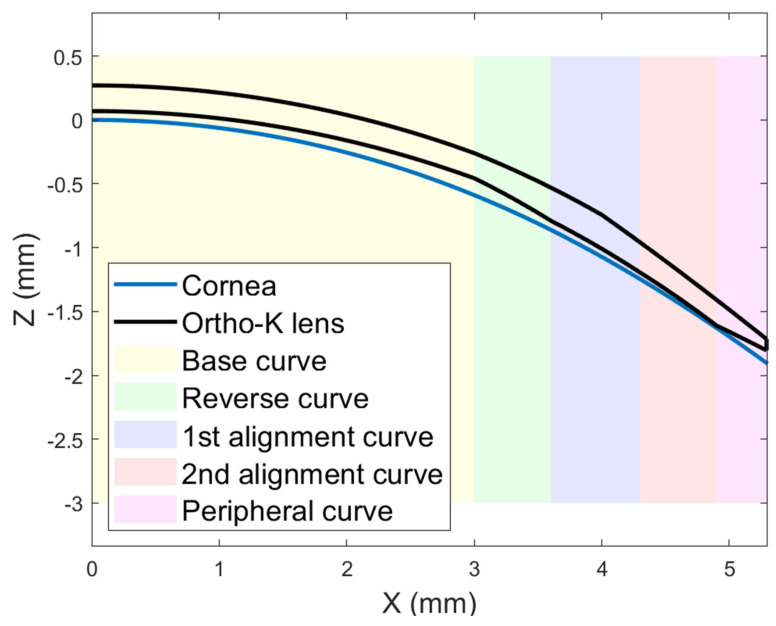
Simplified diagram of a 10.60 mm diameter Ortho-K lens resting on the 18-year-old participant’s right cornea. The lens has a base curve of 8.80 mm, a reverse curve of 6.80 mm, alignment curves of 7.80 mm and 8.00 mm, and a peripheral curve of 11.50 mm. Front curves are 8.70 mm, 8.10 mm, and 4.00 mm from the centre to the edge, respectively. Lens decentration is not considered in this figure for display purposes.

The contact lenses used in this retrospective study were the Hiline design manufactured from Boston XO hexafocon A material (Bausch & Lomb Incorporated, Laval, QC, Canada) with violet handling tint and oxygen permeability (DK) of 100 × 10^−11^ (cm^3^O_2_/cm^2^s), a captive bubble wetting angle of 49°, hardness of 112 Rockwell on the R scale, and a specific gravity of 1.27. The lenses were manufactured by Brighten Optix Corporation (Taipei, Taiwan) using an air-bearing spindle Optoform 40 ophthalmic lathe computer numerical control (CNC) system (Sterling, FL, USA) with a positioning sensing resolution of 8.6 nm. The design comprises four back curvatures, a central base curve (Back Optic Zone Diameter 6.00 mm), a reverse curve, and two successive alignment curves. The centre thickness was 0.22 mm and diameters ranged from 10.00 mm to 10.80 mm with most being 10.60 mm (Figure 1).

### 2.2. Subject Data Collection and Processing

This retrospective study utilised the data from fully anonymised records of 249 eyes fitted with Hiline Ortho-K lenses from which 132 right and 117 left eyes were included, as shown in Table 1. The average age was 14.1 ± 4.0 years (range 9 to 38 years), and all eyes were myopic up to a value of −10.00 DS. The study was approved by the ethical committee of the Institutional Review Board in Taiwan (N201810065) and conducted following the standards set in the Declaration of Helsinki. Only participants with no history of ocular disease, binocular anomaly, trauma, or ocular surgery were included. All participants were selected based on full compliance with the overnight wearing protocol and any non-compliant participants or those who experienced corneal abrasion or other ophthalmic complications were excluded. Exclusions also included any participants who were examined after the early morning period, to avoid ocular changes as the Ortho-K effect wore off.

Topography measurements were carried out with the Medmont E300 instrument. Raw height files “*.hgt” and distance files “*.dst” were digitally scanned and extracted by a custom-built MATLAB code where a grid of 300 angular positions and 333 radial positions were used to construct the corneal anterior surface using the corneal height (raw elevation). Data were read by MATLAB software’s “textscan” function, and the time of examination was established and filtered by reading the “EntryDate” data element from each Medmont E300 *.mxf file along with simulated Keratometry values Flat-K, Steep-K, Flat-Angle, and Steep-Angle where Flat-Angle represented the cylinder axis correction for astigmatism.

### 2.3. Contact Lens Fitting Protocol and Follow-Up

The refractive power of the participants was captured using an autorefractor and checked by subjective refraction with BVA being recorded for initial assessment and post-treatment. The manufacturer’s protocols were followed for fitting and follow-up and all participants were instructed to wear lenses for at least 8 h overnight. IOP and central corneal thickness (CCT) were also recorded at the initial visit using Non-Contact Tono/Pachymeter TONOPACHY™ NT-530P (Nidek Co., LTD., Gamagori, Japan). Participants were followed up at one month and then for regular intervals with the minimum wearing time for the current study being 10 days.

### 2.4. Tangential Refractive Power Change (RPC)

A custom-built MATLAB code calculated tangential refractive power changes following the method described in [17], and then the tangential refractive power change (RPC) was established following the method published in [18], which was also applied in an earlier study [19]. Following this methodology, MATLAB code can produce tangential corneal curvature maps of pre- and post-Ortho-K wear which demonstrate the central flattened zone (CFZ) and annular steepened zone (ASZ) on the treated cornea. The corneal net refractive power $P_{t}$ is typically determined using the reduced Gaussian optics formula [20,21] as

(1) $$P_{t}=\frac{n_{h}-n_{air}}{R_{t}}$$

where the refractive indices of air, $n_{air}$ is set to 1.0, following Gullstrand’s relaxed eye model [22,23]. The anterior radius was set to $R_{t}$ and a hypothetical corneal refractive index of $n_{h}=1.3375$ was used as a correction factor compensating for the absence of the posterior corneal refractive component (Equation (1)), as the Medmont E300 only measures the anterior surface. At this stage, the difference between the corneal post-Ortho-K wear tangential refractive power and pre-Ortho-K wear tangential refractive power, $∆P_{t}$, could then be established as a map according to Equation (2) [19]

(2) $$∆P_{t}={P_{t_{post}}-P}_{t_{pre}}\;\mathrm{where}\;P_{t_{pre}}=\frac{n_{h}-n_{air}}{R_{t_{pre}}}\;\text{\&}\;P_{t_{post}}=\frac{n_{h}-n_{air}}{R_{t_{post}}}$$

**Figure 2:**
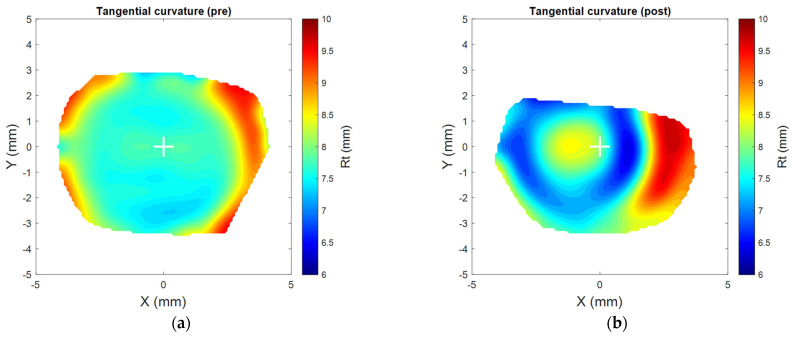
Tangential curvature maps for the right cornea of an 18-year-old participant for pre- (**a**) and post- (**b**) Ortho-K wear. All subsequent figures relate to the same participant’s right eye.

The tangential power maps were smoothed using the robust discretised smoothing spline method [24]. The current study fixed the smoothing factor to 4.5 with tangential maps following [19] to lessen the digital noise affecting the quality of power map illustration and eliminate any edge effect [25], as shown in Figure 2.

Once the power difference map of $∆P_{t}$ was calculated, the CFZ and the ASZ were automatically identified using a technique outlined in [19] which detects pre- and post-wear power profile intersections while avoiding any edge effect [25] by disregarding detected intersections close to the edges of the power map. The central point of the CFZ was then mathematically spotted (see [19] for technical details) and used as an indication of the lens centration compared to the corneal apex. The distance between the centre of the CFZ and the corneal apex was then resolved in X and Y directions using the trigonometric functions cosine and sine respectively and then added to the lens position on the eye during the FEA stage so that any decentration effect was taken into account. This process is essential to ensure that the lens location during the stimulation matches its clinically detected location.

### 2.5. Finite Element Modelling

Analyses were carried out in Abaqus FEA software using the implicit integration scheme, and the nonlinear geometry option “NLGEOM” was active. A custom-built nonparametric meshing method was used, originated by Abass et al. and subsequently implemented in several previous publications [14,15,26,27]. The geometry coordinates were initially converted into spherical coordinates where azimuths, elevations, and radii were used to cubically interpolate the radii of a uniform pre-meshed sphere or hemisphere, as in this study, with a unit radius, and then transformed back to Cartesian coordinates to represent FEA nodes XYZ positions.

The model has three main components: the anterior eye, the Ortho-K lens, and the eyelid. Previous studies that utilised FEA in contact lens–eye models [14,15,26] applied eyelid pressure on the contact lens using a simplified method by introducing a uniformly distributed normal pressure on the lens. For this study, the eyelid was modelled as a full component that applied its pressure through its interaction with the contact lens surface. This allowed a more realistic representation of the eyelid whereby its inner surface had variable contact with the anterior lens surface due to the variation in the front surface design.

FEA was carried out in five steps using an implicit integration scheme, as shown in Table 2. The process started with the stress-free iterations, then inflation of the eye model with the IOP pressure, then a dynamic blink adjusting the lens position before applying the tear surface tension and finally, the closed eyelid pressure on the eyelid component back surface that is facing the eye.

### 2.6. Anterior Eye Model

Medmont E300 height and distance data were used to construct patient-specific FEMs for all participants of the current study; therefore, a total of fully customised 249 FEA models were successfully built and run. As the Medmont E300 coverage area is limited to the central cornea area, a third-order Zernike polynomial [17] was fitted to each eye to reconstruct the surface up to a radius of 5.5 mm [33,34], approximately where the limbus is located [35]. Such a low order Zernike polynomial was used to avoid potential false extrapolation behaviour (i.e., producing a convex shape on edges) that can occur when a higher fitting order is used. In order to maintain the best conceivable level of accuracy, the fitted data within the Medmont E300 measured area were replaced by the original measured surface data, leaving the extrapolated points to cover the lost surface points toward the edges only. In order to achieve a balance between building a full eye model that is time consuming to run and a representative anterior eye model with a lower number of nodes and elements, the averaged eye model developed in [27] was used to extend the anterior eye model 2.5 mm beyond the limbal area. This extension ensured that FEA boundary conditions were not affecting the corneal area where the lens was interacting with the eye.

Due to the lack of posterior corneal measurements from the Medmont E300, this surface was modelled using patient-specific clinically measured CCT. Average and standard deviation values were 0.554 ± 0.033 mm with a range 0.472 to 0.628 mm, in line within the reported ranges in the literature [36,37,38,39], and peripheral corneal thickness was taken to be 0.18 mm thicker than the centre [40,41] with the corneal thickness in between being linearly distributed [27]. Cornea models consisted of 16,400 three-dimensional eight-node solid hybrid elements (C3D8H) with 20,646 nodes in five layers based on a mesh convergence preliminary study. The model edge nodes towards the limbus were restricted as a boundary condition for the simulation.

The anterior eye’s inner wall was identified as an element-based surface, and a core pressure representing the IOP was applied statically in 0.1 equal incremental time steps. The eyes’ stress-free geometry for each participant-specific model at no pressure (IOP = 0 mmHg) was achieved by creating eye models first with the eye’s stressed dimensions. Then, a shirked stress-free geometry of each model was adopted, following the iterative method presented in [28], with the participant-specific measured IOP, which varies among models with an average and standard deviation of 15.02 ± 2.94 mmHg (ranging from 8 mmHg to 24 mmHg). The clinically measured range aligned with known IOP measures [29,42]; therefore, the absolute maximum error was set to a relatively small tolerance of 10^−4^ mm. Corneal material was set to a linear model with a Poisson ratio of 0.49 [36,43] and age-related Young’s moduli set as per [44] (Equation (3)), where the age of participants was automatically calculated via the MATLAB code by subtracting their date of birth from the topography measurement date.

(3) $$E\;(Mpa)=0.0032\;Age\left(years\right)+0.2$$

The corneal and eyelid density as living tissue was set to the water density [13] to allow the performance of the dynamic stage of the simulation during initial blinking.

### 2.7. Eyelid Edge Detection and Modelling

Although topography machines do not measure the eyelid, its footprint can be seen in the Medmont E300’s raw data, where the eyelashes interfere with the topography measurement, resulting in slit-like gaps on the resultant map. Additionally, the eyelid geometry follows the overlayed smoothed-out anterior eye geometry when the eyelid is entirely folded, as appeared in X-ray scans [45] and MRI images [46,47]. As detailed information on how the upper and lower eyelid surfaces interact with and apply pressure to the ocular surface was required, the eyelid’s three-dimensional shape was reverse-engineered from its footprint extracted from the Medmont E300 raw data.

The “slits” seen In the exported topography are usually bridged digitally by the signal processing employed to produce readable topography maps by the Medmont E300 Studio software 7.2.8, hence the need for raw data in the current study. Detecting the eyelash position while the eye was fully open enabled the creation of the eyelid boundary and, thus, the eyelid border geometry. First, the topography’s peripheral boundary was detected; accordingly, the geometrical centre was calculated and then used to determine the radii of all topography border points. The frequency distribution of variable radii was then automatically checked in a histogram with a bin size of 0.3 mm, which was chosen based on preliminary testing and ensuring representation of the range of the examined radii at each rate. The most frequent radii were selected as landmarks of the eyelid edge and its estimated shape. Relevant points were used to fit a second-order curve representing the concavity of the eyelid boundary, as shown in Table 3. The obtained eyelid boundary curve shape was combined with the anterior corneal shape to form an estimated eyelid geometry with a convex shape that matched the anterior eye shape with an average thickness of 0.5 mm [48,49].

**Figure 3:**
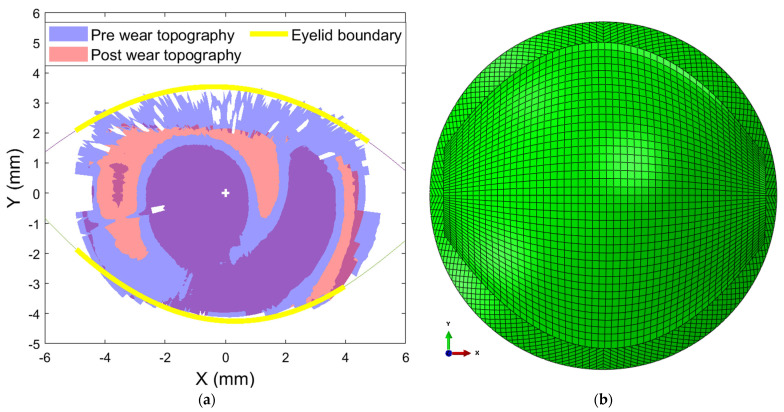
(**a**) Eyelid boundary identification using eyelash footprints detected from eye topography scans for the 18-year-old participant’s right eye. (**b**) Building the FEA model based on eyelash detection, eyelid boundary identification, and eye topography.

As the Ortho-K lenses were effective when the eyelids were closed, both upper and lower eyelids were modelled as one continuous surface. The eyelid surface model’s mesh grid stretched uniformly in 51 steps in the X direction and in 41 steps in the Y direction, in two layers to keep a regular element aspect ratio, where these parameters were selected based on preliminary iterative study exploration, and the odd number of steps ensured having nodes on the X and Y axes for boundary conditions purposes, as shown in Figure 3. Solid hybrid elements of 3999 constructed of eight nodes each (C3D8H) were used in modelling the eyelid with 6272 nodes. An elastic material model was used to model the eyelid with a Young’s modulus of 1.73 MPa and a Poisson ratio of 0.49 [50].

### 2.8. Contact Lens Model

The geometry of each contact lens was precisely constructed based on the rigorous design parameters used to manufacture it. Back surface design information was used to build the base curve, reverse curve, two alignment curves and a peripheral curve with radial orientations, as shown in Figure 1. For the front surface, three curves were used to build a three-dimensional shape. As the lenses are rotationally symmetrical, their cross-section was revolved by sweeping front and back profiles around an axial axis (Z-axis in this case) to construct the three-dimensional closed shape of the lens.

**Figure 4:**
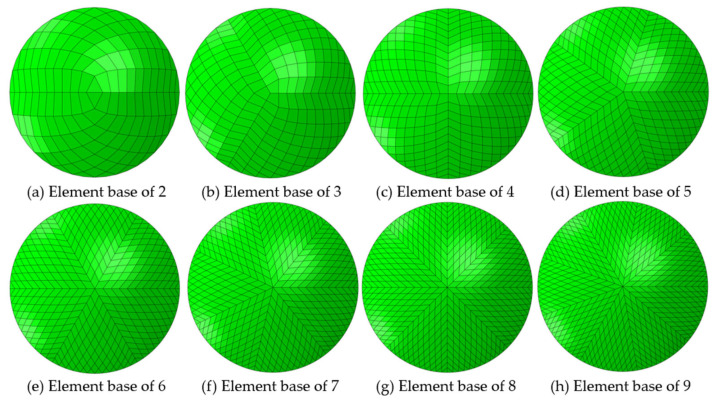
Building the FEA model for Ortho-K contact lenses using different element bases. Subplot (**c**) represents the configuration used in the current study with an element base of 4. The number of rings was kept to 12 in this figure to improve visualisation.

**Figure 5:**
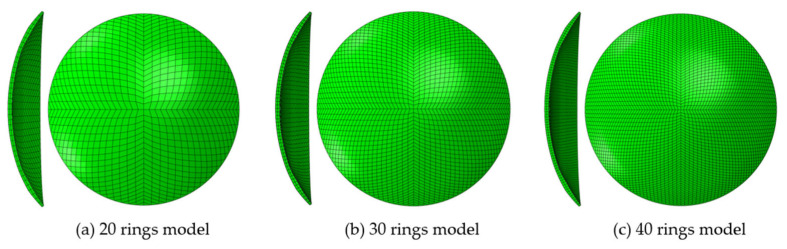
Building the FEA model for Ortho-K contact lenses using different numbers of rings. Subplot (**b**) represents the configuration used in the current study with element base of 4.

The FEA contact lens models were initially built in different element base configurations, as seen in Figure 4, and varying numbers of rings, as in Figure 5, for running a preliminary analysis to select the optimal configuration for creating a patient-specific study. This preliminary optimisation-based investigation led to the selection of a four-element based configuration with 30 rings in two layers.

As corneal topography varies among individuals [51] and no eye has a perfectly spherical cornea [52], lens decentration is not uncommon. In this study, the decentration was calculated by comparing the lens effect on the eye’s RPC and sensing the CFZ’s centre. After applying this detected lens decentration, a process for registering the lens’s back surface against the corneal surface was required. The current study used the iterative closest point (ICP) transformation algorithm on the three-dimensional back surface of the lens to allow the lens to translate and rotate to minimise the normal distance difference between it and the cornea’s anterior surface. Accordingly, the three-dimensional position of the decentred lens model needed fine-tuning to position the lens correctly on the anterior eye surface model. The ICP algorithm was restricted to work with minimal translation and rotation to fine-tune the lens’s back surface on top of the eye through an optimisation loop that prevents the lens’s back surface from penetrating the eye’s anterior surface while minimising the normal distances between the two surfaces. Based on a preliminary investigative study, the optimal number of optimisation iterations was found to be around 20; therefore, the maximum number of ICP iterations was set to 50. Afterwards, the ICP surface registration process produced two matrices representing the translation and the rotation inline and around the three Cartesian coordinates. The Euclidean space rotation matrix R that resulted from the ICP algorithm can be expressed in Equation (4) as

(4) $$R=\left[\begin{array}{ccc} \cos\alpha \cos\beta & \cos\alpha \sin\beta \sin\gamma -\sin\alpha \cos\gamma & \cos\alpha \sin\beta \cos\gamma +\sin\alpha \sin\gamma \\ \sin\alpha \cos\beta & \sin\alpha \sin\beta \sin\gamma +\cos\alpha \cos\gamma & \sin\alpha \sin\beta \cos\gamma -\cos\alpha \sin\gamma \\ -\sin\beta & \cos\beta \sin\gamma & \cos\beta \cos\gamma \end{array}\right]$$

where α is the angle of rotation around the X-axis, β is the angle of rotation around the Y-axis, and γ is the angle of rotation around the Z-axis. Similarly, the translation matrix T can be expressed as in Equation (5) as

(5) $$T=\left[\begin{array}{c} X_{t}\\ Y_{t}\\ Z_{t} \end{array}\right]$$

where $X_{t},\;Y_{t}$ and $Z_{t}$ are the linear translations in X, Y and Z directions, in turn. Hence, the registered or repositioned lens coordinate can be expressed as shown in Equation (6) as

(6) $$\left[\begin{array}{ccc} x_{rt\;1} & x_{rt\;2} & x_{rt\;3}\\ y_{rt\;1} & y_{rt\;2} & y_{rt\;3}\\ z_{rt\;1} & z_{rt\;2} & z_{rt\;3} \end{array}\begin{array}{ccc} & \ldots & x_{rt\;n}\\ & \ldots & y_{rt\;n}\\ & \ldots & z_{rt\;n} \end{array}\right]=R*\left[\begin{array}{ccc} x_{\;1} & x_{\;2} & x_{\;3}\\ y_{\;1} & y_{\;2} & y_{\;3}\\ z_{\;1} & z_{\;2} & z_{\;3} \end{array}\begin{array}{ccc} & \ldots & x_{\;n}\\ & \ldots & y_{\;n}\\ & \ldots & z_{\;n} \end{array}\right]+\left[\begin{array}{c} X_{t}\\ Y_{t}\\ Z_{t} \end{array}\right]$$

where $x_{rt}$, $y_{rt}$ and $z_{rt}$ are the registered coordinates, and x, y and z are the original lens coordinates. The lens’s registered surface is then used to construct the FEA model nodes of the lens.

The properties of fluorosilicone acrylate Boston XO contact lens material were modelled, and the tear layer was simulated by applying a tear fluid surface tension of P_2_ = 43.6 mPa [14,15,32] to the back surface of the contact lens. Each contact lens from the investigated designs was modelled as a linear elastic incompressible solid with a material Young’s modulus of 1500 MPa, a Poisson ratio of 0.49, and 1270 Kg/m^3^ density.

**Figure 6:**
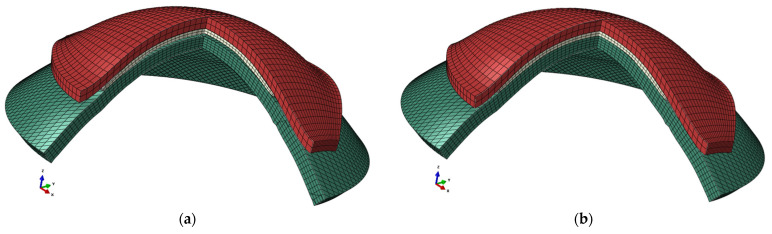
Example of an FEA model for the same 18-year-old participant’s right eye in two stages: (**a**) before applying eyelid pressure and (**b**) after applying eyelid pressure. The eye is displayed in dark green, the lens in light grey and the eyelid in red.

In the settings of Abaqus FEA models, the anterior corneal surface and contact lens’s back surface were taken as master and slave surfaces, respectively. The interaction between these surfaces was further defined by a coefficient of friction of 0.01 [53]. Similarly, the eyelid’s back surface, the lens’s front surface, and the eyelid’s back surface and anterior eye surface are defined as contact surfaces, Figure 6.

### 2.9. Modelling Results Exportation Methodology

**Figure 7:**
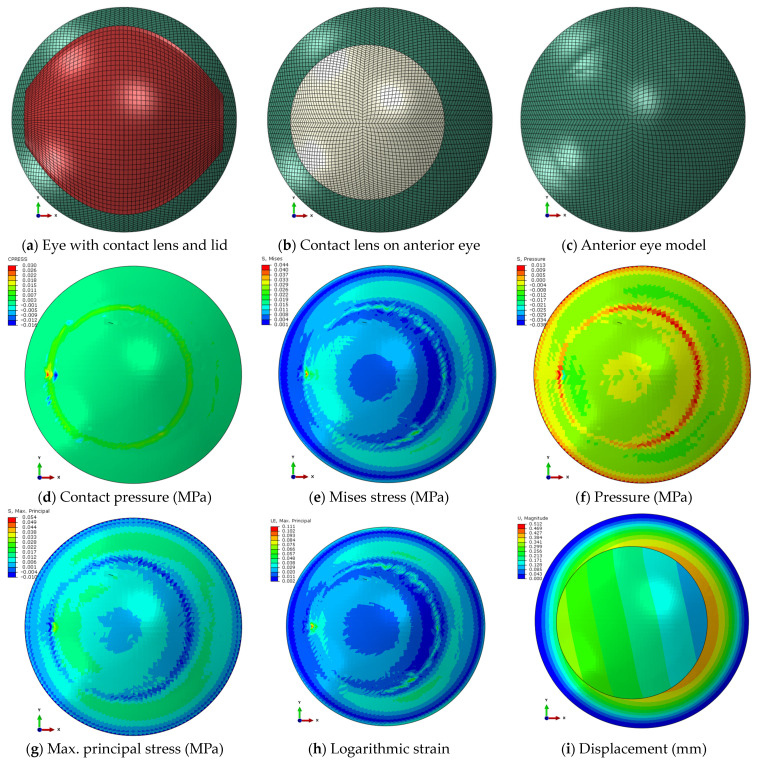
FEA models (**a**–**c**), showing the different stresses (**d**–**g**), strain (**h**) and displacement (**i**) as displayed in Abaqus FEA software for the same 18-year-old participant’s right eye.

**Figure 8:**
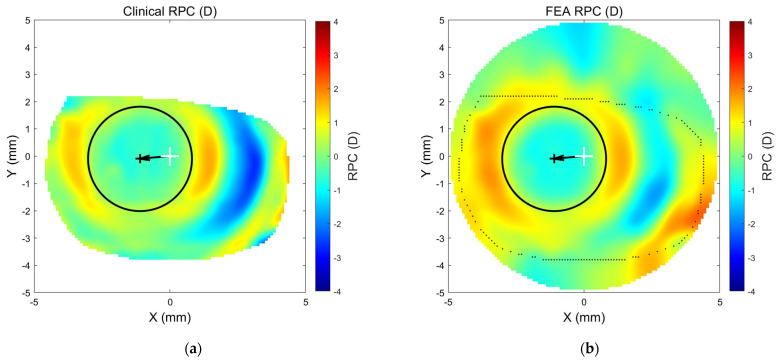
RPC map of for the same 18-year-old participant’s right cornea as measured clinically (**a**) and as determined via patient-specific FEA (**b**).

The results from the Abaqus FEA simulations were exported up to 20 decimals via custom-built Python general-purpose programming language scripts triggered by MATLAB. The Python script output was written in a text file format that contained the node and element numbers with the relevant exported variables and was readable by MATLAB, which was later used for further processing. Anterior eye front nodes, relevant stress–strain components, and their appropriate XY Position were identified. At this stage, displacement, stress, and strain distribution maps like the Abaqus-produced maps in Figure 7could be constructed automatically by MATLAB and compared to the clinical RPC maps (Figure 8) in two ways.

### 2.10. Statistical Analysis

The statistical analysis carried out on the results of this study was performed using the Statistics and Machine Learning Toolbox of the MATLAB software. The null hypothesis, at 95.0% confidence level testing, was used to investigate the differences of the findings based on statistical evidence. The normal distribution of the samples was confirmed using the Kolmogorov–Smirnov test [54]. Then, the two-sample t-test was applied to investigate whether there was significance between pairs of datasets and to verify whether the assessed findings represented an independent record. The probability value (*p*) is an element in the closed period 0.0 to 1.0, where values of *p* higher than 0.05 indicate that the null hypothesis cannot be rejected [55]. In this context, the null hypothesis was that the data in two investigated results represented independent samples.

### 2.11. Two-Dimension (2D) Normalised Cross-Correlation

Once the models had run, a set of custom-built Python scripts exported the nodes’ Cartesian position and their displacements in addition to four stress components: contact pressure, Mises stress, pressure, and maximum principal stress, and one strain component: maximum principal logarithmic strain.

Exporting the Cartesian coordinates of FEA nodes allowed the creation of corneal surface height maps before and after the Ortho-K wear from the simulation results, hence calculating the RPC based on FEA. Maps of the distributions of contact pressure, Mises stress, pressure, maximum principal stress, and one strain component (maximum principal logarithmic strain) were also created based on the exported FEA results. While processing the FEA results, a typical squared mesh grid expanding from −8 mm to 8 mm was created in a step of 0.1 mm as standard XY coordinates for all exported eye node data. Triangulation-based cubic interpolation was used to fit all anterior eyes to the typical grid, allowing the maps to be determined while ensuring common relative positions.

Cross-correlation analyses were conducted to calculate 2D cross-correlation in the spatial frequency domain to identify the essential stress or strain parameters mutually related to the cornea’s RPC resulting from the Ortho-K lens wear. The process was achieved by calculating the local sums via precomputing running sums and using local sums to normalise the 2D cross-correlation to get cross-correlation coefficients $\gamma \left(u,v\right)$ as in Equation (7) [56,57]. RPC was used as the first so-called template with a mean $\bar{t}$ while other maps were used one by one as second matrix $f\left(x,y\right)$ with a mean $\bar{f_{u,v}}$ in the region where it overlapped with the template.

(7) $$\gamma \left(u,v\right)=\frac{\sum_{x,y}\left(f\left(x,y\right)-\bar{f_{u,v}}\right)\left(t\left(x-u,y-v\right)-\bar{t}\right)}{\sqrt{\sum_{x,y}{\left(\left(x,y\right)-\bar{f_{u,v}}\right)}^{2}}\sum_{x,y}{\left(t\left(x-u,y-v\right)-\bar{t}\right)}^{2}}$$

**Figure 9:**
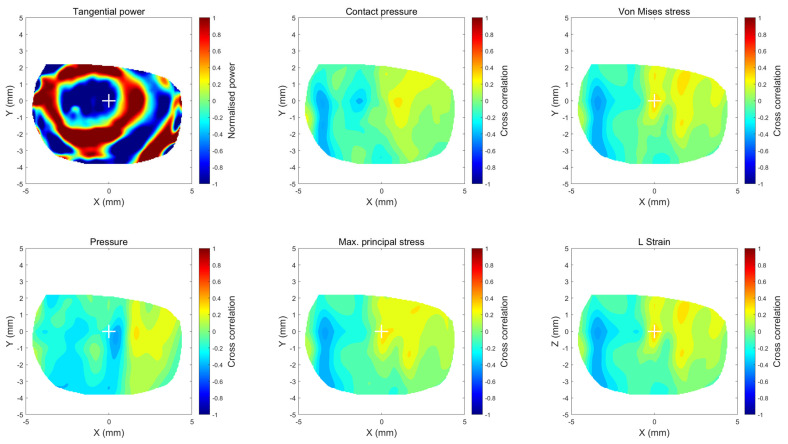
Cross-correlation maps of normalised TPC (first subplot) and the various normalised FEA stress maps in addition to the strain map for the same 18-year-old participant’s right eye.

The 2D cross-correlation coefficient used in this study measures the linear dependence of two variables [58]. Corneal areas with 2D cross-correlation coefficients between −0.4 to 0.4 were considered weak 2D cross-correlation areas, between −0.6, −0.4 or 0.4, 0.6 moderate cross-correlation areas, and −1, 0.6 or 0.6, 1 as strong 2D cross-correlation areas, as shown in Figure 9. As the compared maps have different units and numerical ranges, all maps were normalised within the range −1:1 to ensure a fair comparable comparison. As the study dealt with RPC and stresses as 2D maps, cross-correlation coefficients are represented in maps rather than single values to ensure precise comparison, mainly when considerable Ortho-K decentration occurs.

### 2.12. Structural Similarity (SSIM) Index

The SSIM index is a tool used to assess image quality by comparing a distorted image with a reference image using the degradation of structural information [59]. Since a grayscale image is a matrix with numerical values ranging from 0 to 255 or 0 to 1 when normalised, the principle can be utilised to assess the similarity between two normalised maps, hence systematically indicating the similarity between the normalised RPC map clinically obtained and the normalised FEA outputs. The index gives the scaler value ranging from 1 for perfect similarity, to −1 for perfect inverse similarity, crossing the zero where there is no similarity between the compared matrices. The SSIM index for each $\left(x,y\right)$ point in the map can be expressed as in Equations (8)–(11)

(8) $$SSIM\left(x,y\right)={l\left(x,y\right)}^{\propto }{c\left(x,y\right)}^{\beta }{s\left(x,y\right)}^{\gamma }$$

(9) $$\mathrm{with}\;\;\;l\left(x,y\right)=\frac{2\mu_{x}\mu_{y}+C_{1}}{{\mu_{x}}^{2}+{\mu_{y}}^{2}+C_{1}}$$

(10) $$c\left(x,y\right)=\frac{2\sigma_{x}\sigma_{y}+C_{2}}{{\sigma_{x}}^{2}+{\sigma_{y}}^{2}+C_{2}}$$

(11) $$s\left(x,y\right)=\frac{\sigma_{xy}+C_{3}}{\sigma_{xy}+C_{3}}$$

where $\mu_{x}$ and $\mu_{y}$ are the local means, $\sigma_{x}$ and $\sigma_{y}$ are the local standard deviations and $\sigma_{xy}$ is the cross-covariance for the matrices x,y, and $C_{1}$, $C_{2}$, and $C_{3}$ are the regularisation constants for the luminance. Following the linear dependency measure in [58] and to allow parallel valuation with 2D cross-correlation coefficients, corneal areas with an SSIM index between −0.4 to 0.4 were regarded as weak similarity areas, between −0.6, −0.4 or 0.4, 0.6 moderate similarity areas and −1, 0.6 or 0.6, 1 as strong similarity areas. SSIM indices were represented in the same way as 2D cross-correlation coefficients in maps rather than single averaged values to ensure precise comparison, especially when Ortho-K decentration happened, and single averaged values became unreliable.

## 3. Results

Once the 249 participant-specific modelling processes were created, the FEA revealed variation in the distribution of the different stress and strain components on the anterior eye model, as seen in the example of an 18-year-old participant in Figure 8.

The study’s choice of main parameters was confirmed through preliminary analyses of the FEA compared to the clinical dataset RPC to examine individual variables’ distributions. As in the example, in the case displayed in Figure 7, FEA was performed with eyelid pressure of 8 mmHg, 12 mmHg and 16 mmHg and recorded RMSs of 0.1102 D, 0.1452 D, and 0.1454 D, confirming the value of 8 mmHg as the best choice.

As the clinically detected lens decentration was considered in the modelling process, and using measured eye topography with their asymmetric properties, if any, to build models, representative decentred stress and strain distributions were obtained where the decentred effect could be noticed visually and detected numerically. Visual inspection of the Ortho-K lens’s footprint on the maps showed that Mises stress (Figure 7e), pressure (Figure 7f), maximum principal stress (Figure 7g), and logarithmic strain (Figure 7h) displayed distributions sensibly linked to clinically measured RPC; however, contact pressure distribution was relatively concentrated around the periphery of the Ortho-K lens with a limited footprint on the central area (Figure 7d). Cross-correlation and SSIM index maps of normalised RPC and FEA normalised stress maps and strain maps were the quantifiable evaluation of the best correlated FEA component to the clinically measured RPC.

**Figure 10:**
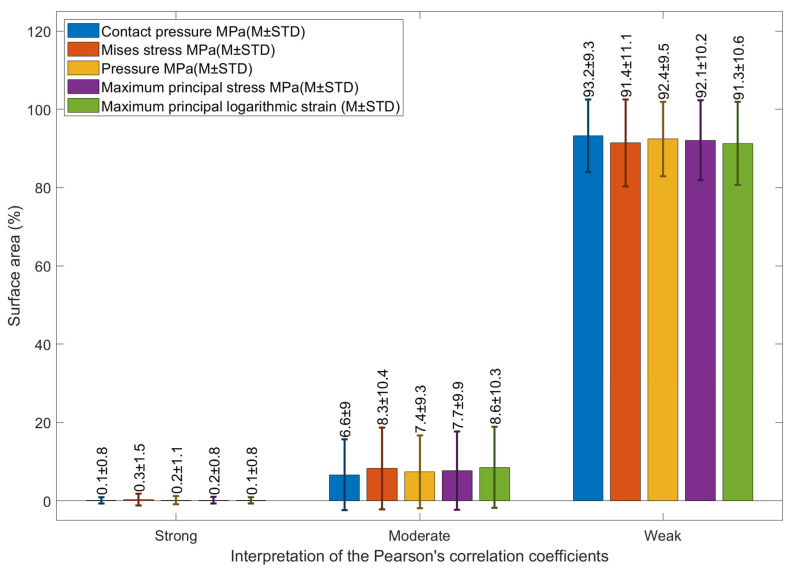
Using the combined data for all eyes in the study, this figure shows the percentage of corneal area that cross-correlated with the clinically measured EPC. Pearson’s correlation coefficients were used to classify the degree of correlation as strong, moderate and weak.

On the one hand, when corneal areas were classified as strong, moderate and weak according to their 2D cross-correlation coefficients in Figure 9, none of the stress or strain components recorded a strong 2D cross-correlation area with the clinical RPC (Figure 10); however, when it came to moderate 2D cross-correlation, maximum principal logarithmic strain recorded the highest area with 8.6 ± 10.3%, and contact pressure recorded the lowest area of 6.6 ± 9%, while Mises stress recoded second with 8.3 ± 10.4%. Contact pressure recorded the highest weak 2D cross-correlation area of 93.2 ± 9.3% on the weak 2D cross-correlation bar.

**Figure 11:**
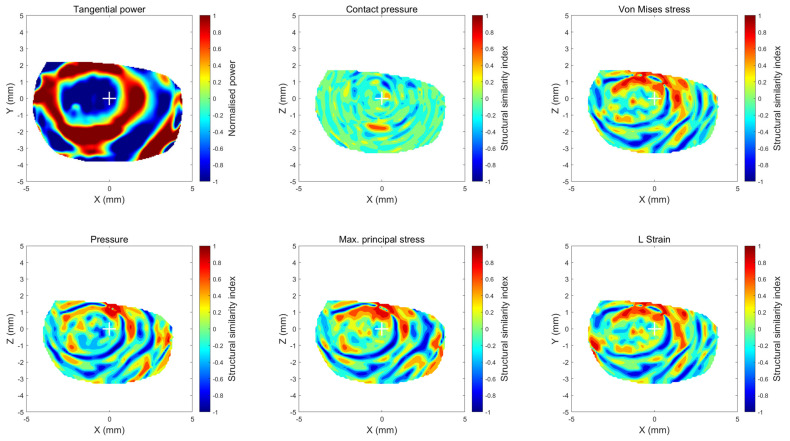
Using the same right eye of the 18-year-old participant, this figure shows the structural similarity index (SSIM) maps. The first subplot is the normalised tangential power map and 4 FEA normalised stress maps in addition to the strain map.

**Figure 12:**
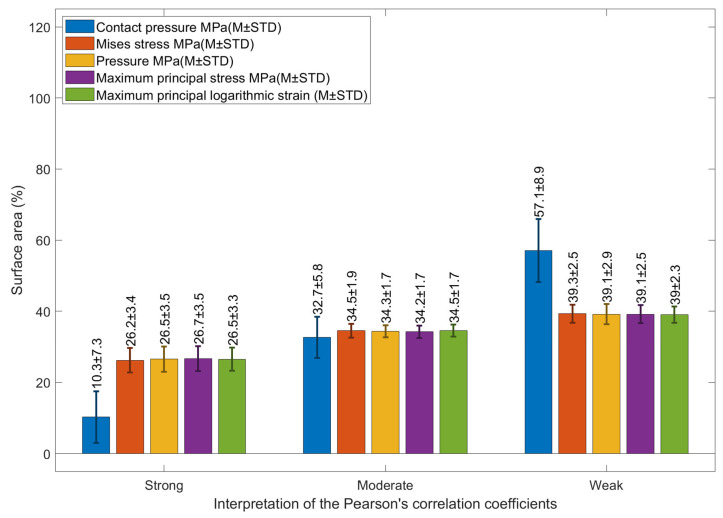
Percentage of corneal areas that had a structural similarity index (SSIM) with the clinically measured EPC where Pearson’s coefficient ranges were used to classify the degree of similarity as strong, moderate and weak.

On the other hand, when the similarity measure SSIM index was used to compare the areas that were most similar to the clinical RPC (Figure 11), maximum principal stress was the most similar with an average strong similarity percentage area of 26.5 ± 3.3% (Figure 12); and contact pressure was the least strong similarity area of 10.3 ± 7.3%. Regarding the moderate similarity areas, all components were recorded at around 34.4% similarity area except contact pressure, which was down to 32.7 ± 5.8%. Again, larger weak similarity areas were associated with the contact pressure with 57.1 ± 8.9% using the SSIM index.

## 4. Discussion

Prescribing Ortho-K contact lenses is still considered a niche activity, although it is increasingly being used because of the interest in its myopia control properties [60] in addition to myopia management [61,62,63,64,65]; therefore, the research into the engineering aspects of its working mechanism is still limited. The RPC in Ortho-K, believed to be achieved by epithelial thinning in the central cornea and thickening towards the periphery, has associated biological and mechanical risks. Clinically, there is a risk of reducing resistance to microbial infection by weakening the central epithelial barrier [9,10,66,67,68,69]. From the mechanical point of view, lens–eye interactions can result in variable visual results due to residual refractive error [70].

The ability to predict outcomes for individuals from Ortho-K treatment based on initial examination and measurements of the patient is important, not only for refining myopia management and the RPC [13,14,15], but also for minimising ocular complications, improving first fit success rates and reducing chair time. Simulation of on-eye contact lens performance using Finite Element Analysis (FEA) provides a methodology whereby ocular measurements and attributes together with contact lens design and material properties can be incorporated into a model of the lens–eye interaction. However, to build effective models, it is first necessary to identify the most useful clinical, material and mechanical metrics; that is, those that have the greatest impact on the model in terms of accuracy. This, then, influences which ocular measurements should be taken during the examination and which FEA components are chosen for modelling.

Clinically, the eyelid is the most difficult to quantify in terms of position, blink rate and the force it applies to the lens and eye. This study applied a novel eyelid boundary edge detection that enabled the FEA model to mimic the effect of the eyelid pressure on both the lens and eye rather than only applying the pressure normally on the contact lens front surface as in previous work [14,15,16]. The previous method of eyelid representation was a limitation, as the eyelid pressure direction followed the contact lens’s front surface geometry normally rather than the eyelid’s back surface geometry. This new study is a major update in how the contact lens’s interaction with the eye is simulated and allows the capture of eyelid stresses on the eye in the areas sounding the contact lens. Equally, it shows the performance of both centres and edges of the contact lenses on eyes precisely using FEA.

In terms of FEA components, areas of stress concentration are important to identify potential complications of corneal staining or abrasion. To identify the optimal stress components, maps of stress components representing contact pressure, Mises stress, pressure, maximum principal stress, and strain components representing maximum principal logarithmic strain were examined against the clinically obtained RPC in two ways: firstly, by 2D cross-correlation and then via the SSIM index. The study built and processed 249 patient-specific FEA models backed with clinical data and, for the first time in the FEA investigation of Ortho-K fitting, the lens decentration was considered within the model. Unlike previous theoretical approaches, which assumed the lens was perfectly centred and the stress and strain distributions were rotationally symmetrical [17,71], the actual lens position was reflected in the strain and stress distribution. The study showed that comparing the lens’s geometry parameters to the stress distributions indicates that the Ortho-K footprint on the eye is complicated because of the decentration frequently associated with its performance. Considering that perfect centration is very challenging to achieve, simulating Ortho-K lenses ignoring the decentration could lead to misinformed results that cannot be used accurately to achieve patient-specific results.

The study also applied a novel eyelid boundary edge detection that enabled mimicking the effect of the eyelid pressure on both the lens and eye rather than only applying the pressure normally on the contact lens’s front surface as in previous work [13,14,15]. This method in previous work was a limitation, as the eyelid pressure direction followed the contact lens’s front surface geometry rather than the eyelid’s back surface geometry. This new study is a major update in how the contact lens’s interaction with the eye is simulated and allows the capture of eyelid stresses on the eye in the areas sounding the contact lens. Equally, it shows the performance of both centres and edges of the contact lenses on eyes precisely using FEA.

Different FEA stress components, such as contact pressure, Mises stress, pressure, maximum principal stress, and logarithmic strain showed that some FEA components are more sensitive than others to the RPC detected clinically. Maximum principal stress was the most representative FEA component as it showed a close trend to the RPC within the CFZ and the PSZ with positive stress on the centre and negative stress towards the periphery as expected. This is because the reverse curve creates a gap between the back of the lens and the corneal surface which is filled with tears. The combination of central positive and peripheral negative pressures forces epithelial cells to migrate from the central zone to the annular peripheral zone [2,9,10]. The study indicates that if Mises stress, pressures, maximum principal stress or logarithmic strain are used as potential metrics, the outcomes correlate well with RPC or give a high similarity index to RPC distribution. When considering strain, the maximum principal logarithmic strain was an effective barometer as it recorded similar 2D-cross-correlations and similarity indices to maximum principal stress.

Interestingly, the study showed that FEA contact pressure was the worst possible metric for detecting RPC, with the worst 2D cross-correlation and similarity index to RPC. The main reason for this outcome is that the FEA contact pressure component, as it appears from the name, was built on FEA contact surface algorithms; therefore, when applied to reverse geometry Ortho-K lenses, where there are areas of negative pressure, this component cannot properly correlate with the ASZ, which is important in forming the overall RPC effect. It was also noticed that contact pressure distribution was not as sensitive to lens decentration, compared to other stress and strain components, as it only deals with contact between surfaces.

Our study confirmed that FEA-based contact pressure is relatively noisy unless a very intense mesh is used which dramatically increases the computational time. As reported in the Abaqus FEA software manual, the Abaqus software applies built-in smoothing techniques for second-order surfaces to reduce this noise. This suggests why, if the surface is not smooth enough to be approximated to a second-order surface, as in the case where different back lens surface curves meet, many scattered noisy disturbances were noted in the contact pressure distribution compared to other components. In addition, the corneal anterior surface may require order 12 polynomials to be adequately fitted [18].

In conclusion, FEA is an increasingly effective tool in being able to predict the refractive outcome of Ortho-K treatment. Its accuracy depends on identifying which clinical and modelling metrics contribute to the most accurate prediction of RPC with minimal ocular complications.

In terms of clinical metrics, age, IOP, CCT, surface topography and the 3D eyelid effect are all important for effective modelling. A limitation of the study was that lens decentration could not be automatically predicted with a high success rate due to limited troubleshooting data, but this will be addressed in a separate study. As more experience in modelling is gathered from troubleshooting Ortho-K fits in the future, it may be possible to predict decentration as well, thus improving the value of FEA use in clinical settings.

In terms of FEA components, maximum principal stress was found to be the best FEA barometer that can be used to predict the performance of Ortho-K lenses as it provided the best 2D cross-correlations and similarity indices to corneal RPC. In contrast, contact pressure provided the worst performance. In terms of strain, the maximum principal logarithmic strain was an effective barometer as it recoded very similar 2D cross-correlations and similarity indices to maximum principal stress.

## Figures and Tables

**Table 1:** Study participant averaged data.

Time of Wear (Days)	Number of Eyes	Age in Years (m ± std)	IOP (mmHg)	CCT (µm)	Pre Simulated Keratometry (Sim-K) (D)		Post Simulated Keratometry (Sim-K) (D)		Pre-Wear Asphericity		Post-Wear Asphericity		Eye	
					Flat (m ± std)	Steep (m ± std)	Flat (m ± std)	Steep (m ± std)	Flat (m ± std)	Steep (m ± std)	Flat (m ± std)	Steep (m ± std)	Right	Left
10 to 100	249	14.1 ± 4	15 ± 3	554 ± 33	42.6 ± 1.3	44 ± 1.3	41 ± 1.3	42.7 ± 1.5	0.66 ± 0.1	0.4 ± 0.18	0.34 ± 0.14	0.43 ± 0.17	132	117

**Table 2:** Finite element simulation parameters.

Model	Step	Description	Integration Scheme	Loading Condition	Time
Eye	1	Stress-free iterations [28]	Implicit	Static	Normalised increments (0:1)
	2	Inflation by patient-specific IOP [29]	Implicit	Static	Normalised increments (0:1)
Lens between eye and eyelid	3	Eyelid blinking pressure 8.0 mmHg [30]	Implicit	Dynamic	0.6 s, see [31]
	4	Surface tension 43.6 mPa [32]	Implicit	Static	Normalised increments (0:1)
	5	Eyelid closer pressure 8.0 mmHg [30]	Implicit	Static	Normalised increments (0:1)

**Table 3:** Second-order eyelid fitting parameters.

$y=ax^{2}+bx+c$		a (m ± std)	b (m ± std)	c (m ± std)
Right eyes	Upper eyelid	−0.0832 ± 0.0197	−0.0262 ± 0.0445	3.5809 ± 0.4375
	Lower eyelid	0.0842 ± 0.0197	0.0163 ± 0.0445	−3.9734 ± 0.4375
Left eyes	Upper eyelid	−0.0864 ± 0.0187	0.0256 ± 0.0463	3.6564 ± 0.3865
	Lower eyelid	0.0868 ± 0.0187	−0.0206 ± 0.0463	−3.9902 ± 0.3865

## Data Availability

The data presented in this study are available on request from the corresponding author.
